# Rapid prediction of MRI-induced RF heating of active implantable medical devices using machine learning

**DOI:** 10.1109/EMBC40787.2023.10340900

**Published:** 2023-07

**Authors:** Jasmine Vu, Pia Sanpitak, Bhumi Bhusal, Fuchang Jiang, Laleh Golestanirad

**Affiliations:** Department of Biomedical Engineering, McCormick School of Engineering, Northwestern University, Evanston, IL, USA; Department of Radiology, Feinberg School of Medicine, Northwestern University, Chicago, IL, USA; Department of Radiology, Feinberg School of Medicine, Northwestern University, Chicago, IL, USA; Department of Radiology, Feinberg School of Medicine, Northwestern University, Chicago, IL, USA; Department of Biomedical Engineering, McCormick School of Engineering, Northwestern University, Evanston, IL, USA; Department of Radiology, Feinberg School of Medicine, Northwestern University, Chicago, IL, USA; Department of Biomedical Engineering, McCormick School of Engineering, Northwestern University, Evanston, IL, USA; Department of Radiology, Feinberg School of Medicine, Northwestern University, Chicago, IL, USA

## Abstract

The interaction between an active implantable medical device and magnetic resonance imaging (MRI) radiofrequency (RF) fields can cause excessive tissue heating. Existing methods for predicting RF heating in the presence of an implant rely on either extensive phantom experiments or electromagnetic (EM) simulations with varying degrees of approximation of the MR environment, the patient, or the implant. On the contrary, fast MR thermometry techniques can provide a reliable real-time map of temperature rise in the tissue in the vicinity of conductive implants. In this proof-of-concept study, we examined whether a machine learning (ML) based model could predict the temperature increase in the tissue near the tip of an implanted lead after several minutes of RF exposure based on only a few seconds of experimentally measured temperature values. We performed phantom experiments with a commercial deep brain stimulation (DBS) system to train a fully connected feedforward neural network (NN) to predict temperature rise after ~3 minutes of scanning at a 3 T scanner using only data from the first 5 seconds. The NN effectively predicted ΔT_max_—R^2^ = 0.99 for predictions in the test dataset. Our model also showed potential in predicting RF heating for other various scenarios, including a DBS system at a different field strength (1.5 T MRI, R^2^ = 0.87), different field polarization (1.2 T vertical MRI, R^2^ = 0.79), and an unseen implant (cardiac leads at 1.5 T MRI, R^2^ = 0.91). Our results indicate great potential for the application of ML in combination with fast MR thermometry techniques for rapid prediction of RF heating for implants in various MR environments.

## I. Introduction

Magnetic resonance imaging (MRI) is the preferred imaging modality for numerous neurological and cardiac disorders. Despite continuous advances in MRI, a persistent safety concern for patients with active implantable medical devices (AIMDs), such as deep brain stimulation (DBS) systems and cardiac implantable electronic devices (CIEDs), is the risk of radiofrequency (RF) heating of the tissue surrounding the conductive lead [1]. Therefore, there have been consistent efforts to quantify and reduce RF heating of AIMDs. The technical specification ISO/TS 10974, recognized by the FDA as the consensus standard for MR-Conditional devices, outlines a four-tier approach to quantify RF heating. For elongated implants, such as leads in neuromodulation or cardiac devices, recommended test procedures include application of the transfer function [2] or full-wave electromagnetic (EM) simulations of the patient body model and the medical implant. However, there are instances when these standard methods are not feasible, such as in the presence of abandoned or broken leads, either alone or near intact leads [3]. Another challenge is that the recommended limits set by device manufacturers to ensure the safety of the general population may be too conservative for specific cases. For example, RF heating of leads in neuromodulation and cardiac electronic implants has been shown to vary greatly between patients, with up to two orders of magnitude difference, due to the lead trajectory [4]-[8]. Thus, a tool that can predict RF heating around the tip of an unknown lead in an individual, based on the heating profile in the first few seconds of a standard scan, would be highly desirable.

Recent research has employed machine learning (ML) to predict subject-specific local specific absorption rate (SAR) from B_1_^+^ maps [9] and SAR distributions from anatomical MRI images [10]. Neural networks (NNs) have also been utilized to predict SAR during MRI of orthopedic fixation plates based on their geometric features [11], [12]. Our group has previously trained a NN to predict trajectory-specific SAR of DBS leads using the distribution of the tangential component of the incident electric field along each lead trajectory [1], [13]. However, to date, no studies have attempted to predict local temperature changes using ML.

In this study, we present a ML-based algorithm that predicts temperature rise in the tissue surrounding the tips of various AIMD leads after ~3 minutes of RF exposure during MRI scans at 1.2 T, 1.5 T, and 3 T, from only the heating profile in the first 5 seconds of each scan. The model was trained using data from a full commercial DBS system implanted in a tissue-mimicking phantom undergoing MRI at 3 T. Subsequently, we evaluated the generalizability of the resulting neural network architecture by testing its ability to predict the RF heating of a DBS system during MRI scans at a different field strength (1.5 T), as well as a different field strength and polarization (1.2 T vertical scanner), and for unseen leads (cardiac pacemaker systems undergoing 1.5 T MRI). This proof-of-concept work paves the way for developing a clinically applicable tool to predict RF heating of unknown leads during MRI scans.

## II. Methods

### A. Dataset

**Figure 1.**
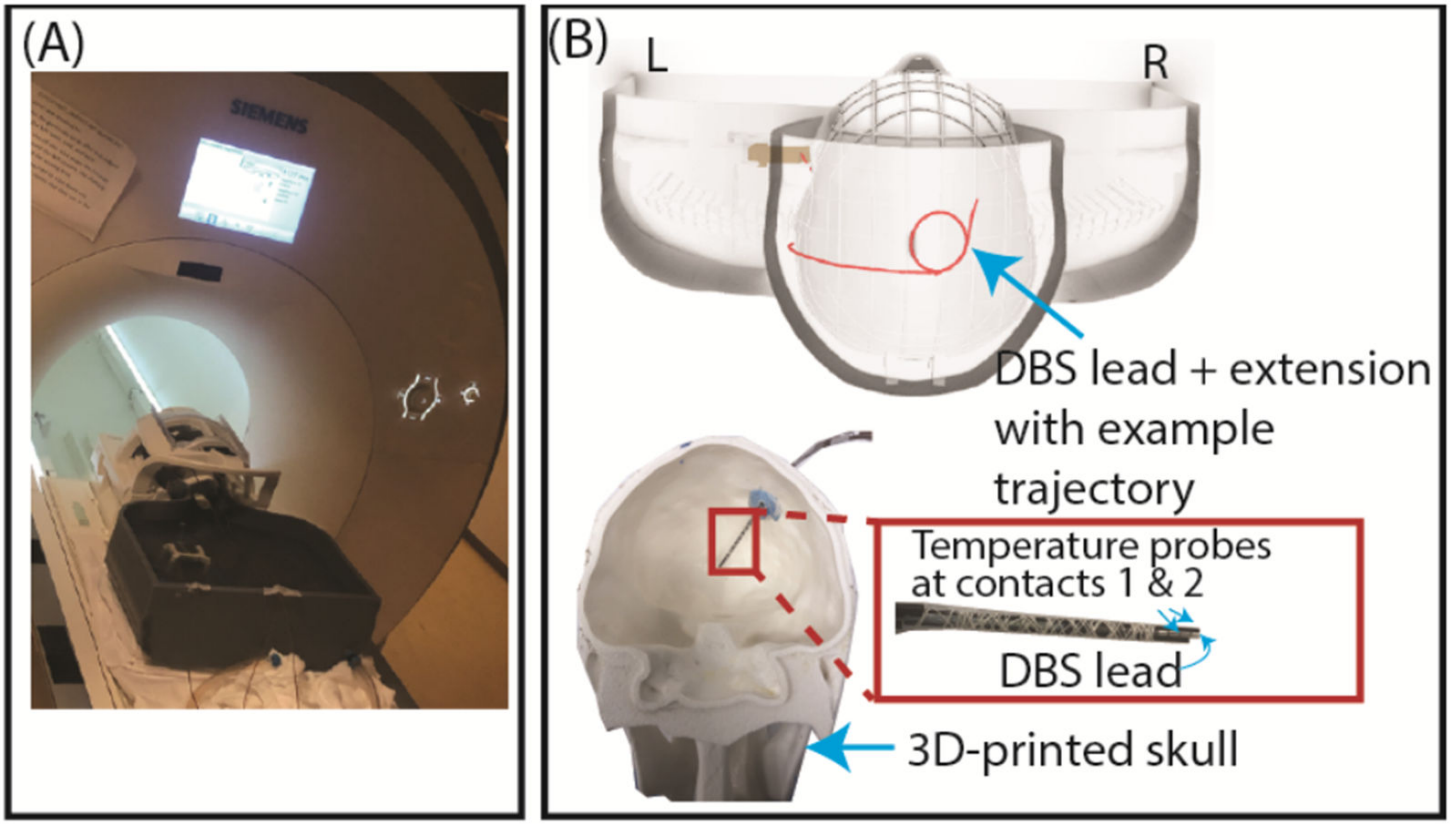
(A) Example experimental setup at a 3 T Siemens Prisma scanner. (B) 3D rendering of an anthropomorphic phantom with a DBS system. Fiber optic temperature probes attached to the DBS lead were used to measure the temperature at the lead-tip.

The dataset consisted of 346 temperature measurements obtained from RF heating experiments performed with a full DBS system from Abbott, including a 40 cm lead (model 6172), a 50 cm extension (model 6371), and an Infinity-5 IPG implanted in an adult-sized anthropomorphic phantom with 346 distinct trajectories (as shown in Fig. 1). The RF exposure was generated using a high-SAR pulse sequence (B_1_^+^rms = 2.7 μT) in a 3 T Siemens Prisma scanner (operating at 123 MHz) (Siemens Healthineers, Erlangen, Germany). The maximum temperature rise (ΔT_max_) in the surrounding gel was recorded using fiber-optic temperature probes (Osensa, Burnaby, BC, Canada, with a resolution of 0.01 °C) attached to the lead tips. The temperature was recorded at 0.5-second increments throughout the RF exposure, which lasted for a total of 150 seconds. The ΔT_max_ values were used to train the ML algorithm and served as the intended output. To ensure consistency across training, validation, and testing of the ML algorithm, ΔT_max_ was shifted such that the initial temperature—the starting room temperature of the phantom gel varied from 20-24 °C—was set to 0 °C for all experimental configurations.

### B. Feature Selection

The inputs to the NN were the measured temperature values during the first five seconds of RF exposure. The inputs consisted of 11 temperature values, starting from 0 °C with increments of 0.5 °C. The first five seconds were selected as the inputs as the goal was to predict ΔT_max_ around the tip of an implanted lead as quickly as possible.

### C. Artificial Neural Network Architecture

The total dataset was divided into the training/validation dataset (70%) and hold-out test dataset (30%). The NN was implemented in Python (3.9.13) using Keras (2.11.0) with Tensorflow as the backend. The performance of the NN was evaluated based on R-squared (R^2^) and the mean squared error (MSE).

Hyperparameter optimization was performed to determine the optimal architecture of the NN (i.e., the number of hidden layers and hidden neurons per hidden layer). This was performed using GridSearchCV with three-fold cross-validation from the scikit-learn package (1.0.2) in Python. The optimal parameters were selected based on the NN model that had the lowest MSE.

**Figure 2.**
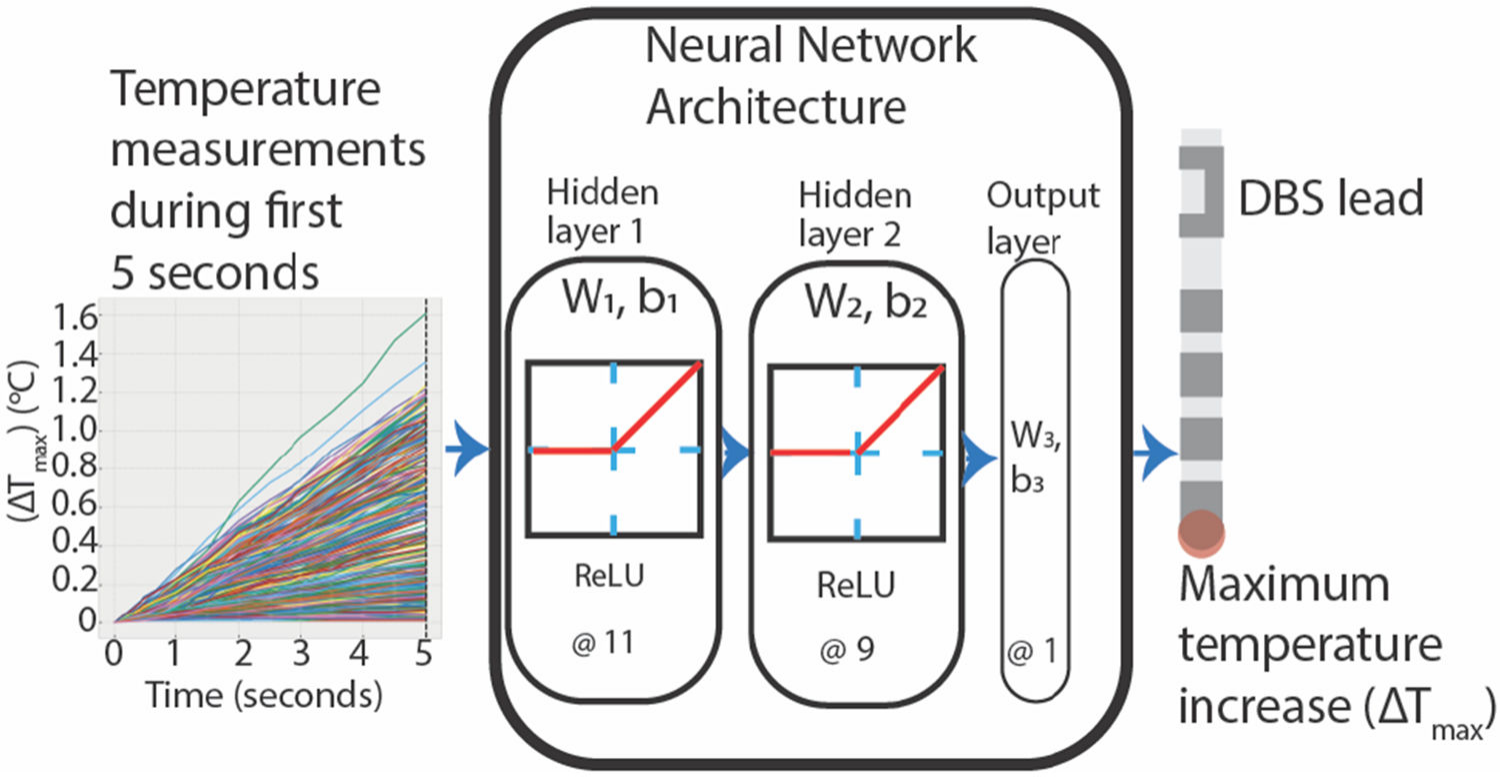
Architecture of the final neural network consisting of two hidden layers with 11 and 9 hidden neurons in the first and second layer, respectively. The first five seconds of experimentally measured temperature values served as the inputs to predict the maximum temperature rise.

The final ML algorithm consisted of a feedforward NN with one input layer, two fully connected hidden layers with 11 and 9 hidden neurons, respectively, and an output layer (Fig. 2). The activation function in the hidden layers was rectified linear unit (ReLU) and linear in the output layer. The Adam optimization routine determined the network weights, and other model parameters included a learning rate of 0.01 and a batch size of 32. The output of the NN was the predicted ΔT_max_, which was then compared to the actual ΔT_max_ from the RF heating experiments.

### D. Generalizability of Neural Network

To test the generalizability of the NN, we used three additional datasets from RF heating experiments with different AIMDs during MRI at different Larmor frequencies. These datasets included results from a DBS system during 1.5 T MRI (Siemens Aera closed-bore scanner, 63.6 MHz) with 30 measurements, a DBS system during 1.2 T MRI (Fujifilm Oasis open-bore scanner, 50.4 MHz) with 28 measurements, and cardiac pacemaker systems from Medtronic (Azure^™^ XT DR MRI SureScan^™^ IPG with a CapSure^®^ EPI lead 4965-15 cm or lead 4965-25 cm) during 1.5 T MRI with 75 measurements. The NN algorithm was applied to these new datasets to evaluate its ability to predict the RF heating of different AIMDs under different MRI conditions.

## III. Results

### A. Distribution of Experimental Temperature Increase

**Figure 3.**
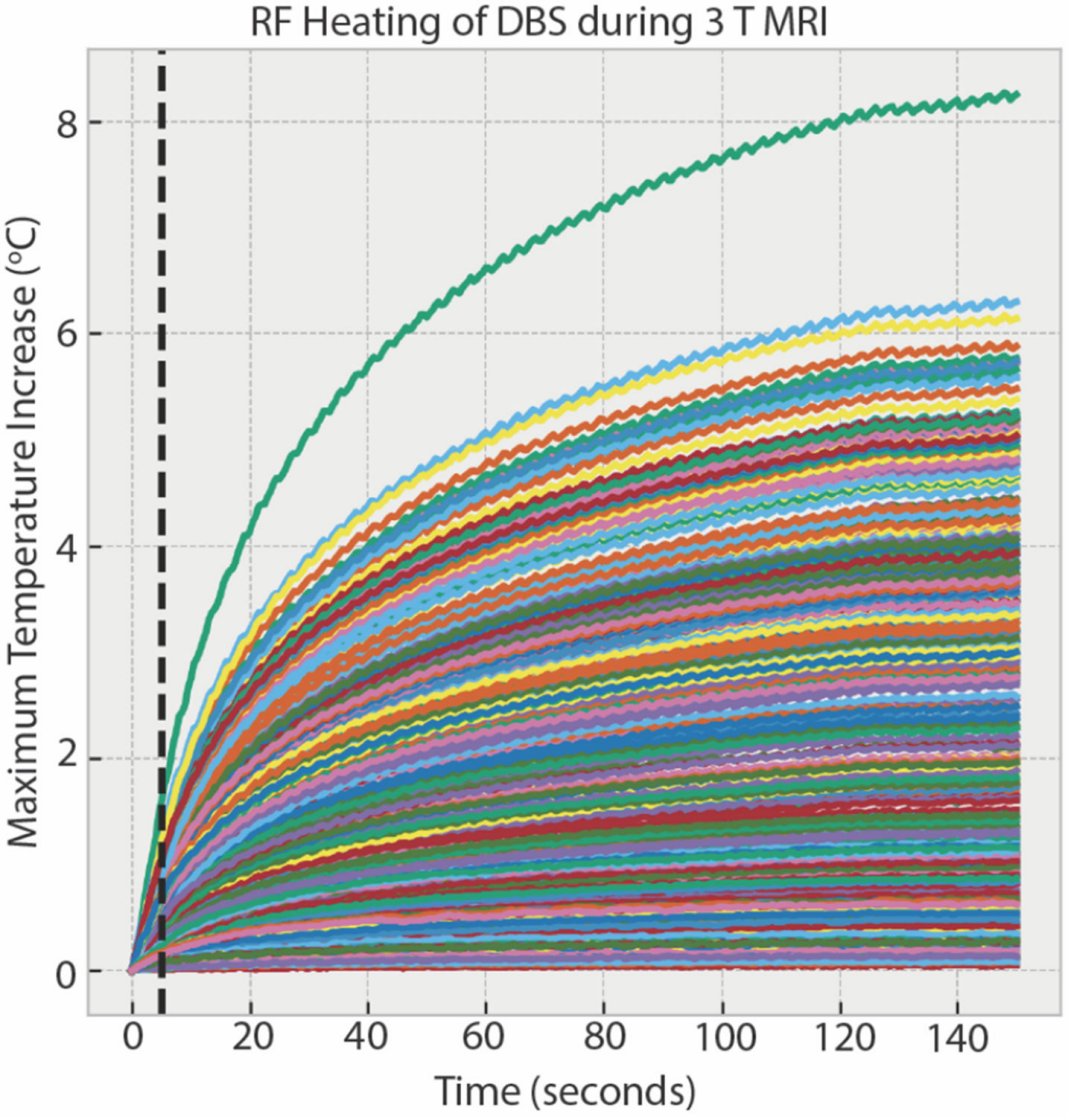
Temporal profiles of temperature at the DBS lead-tip during RF exposure for the 346 different DBS configurations. The black dotted line indicates the temperature after five seconds of RF exposure.

**Figure 4.**
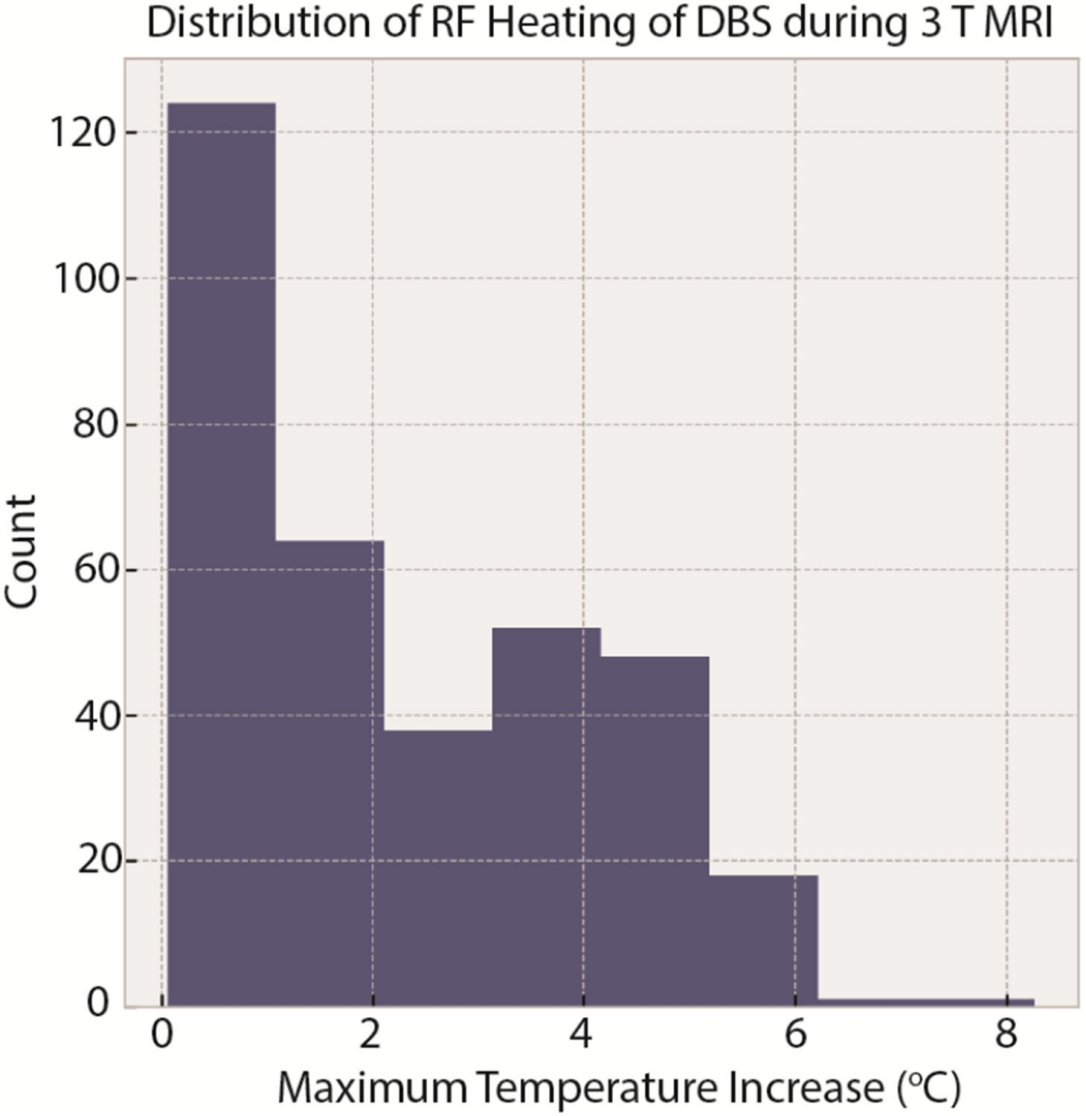
Distribution of ΔT_max_ for 346 phantom experiments performed with a commercial DBS system.

**Figure 5.**
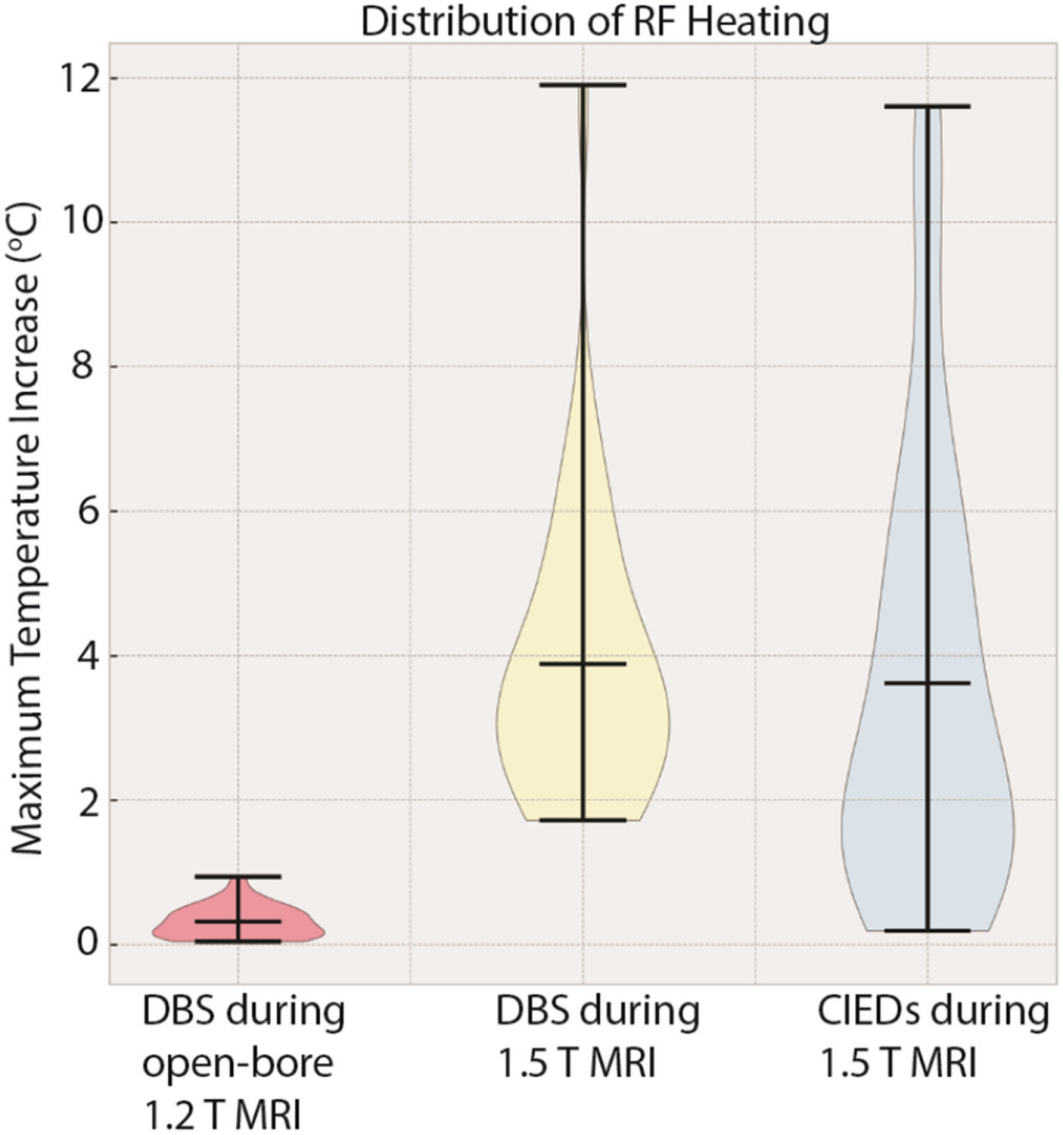
Distribution of ΔT_max_ for cases in the additional test datasets including RF heating of a DBS system during MRI in an open-bore scanner and MRI at 1.5 T and of CIEDs during MRI at 1.5 T.

The RF heating data for 346 configurations in the initial dataset showed a mean ± standard deviation of ΔT_max_ of 2.29 ± 1.76 °C, with a range of 0.05-8.27 °C. Similarly, for the DBS system during 1.2 T MRI, the mean ± standard deviation of ΔT_max_ was 0.31 ± 0.23 °C with a range of 0.04-0.94 °C. during During 1.5 T MRI, the mean ± standard deviation of ΔT_max_ was 3.88 ± 2.11 °C with a range of 1.72-11.90 °C. The cardiac pacemaker systems had a mean ± standard deviation of ΔT_max_ of 3.62 ± 3.10 °C with a range of 0.19-11.6 °C. Fig. 3 shows the temporal profile of ΔT_max_ for the 346 configurations in the initial dataset, and Figs. 4 and 5 show the distribution of ΔT_max_ for these four unique datasets.

### B. Effectiveness of ANN-based Predictions

**Figure 6.**
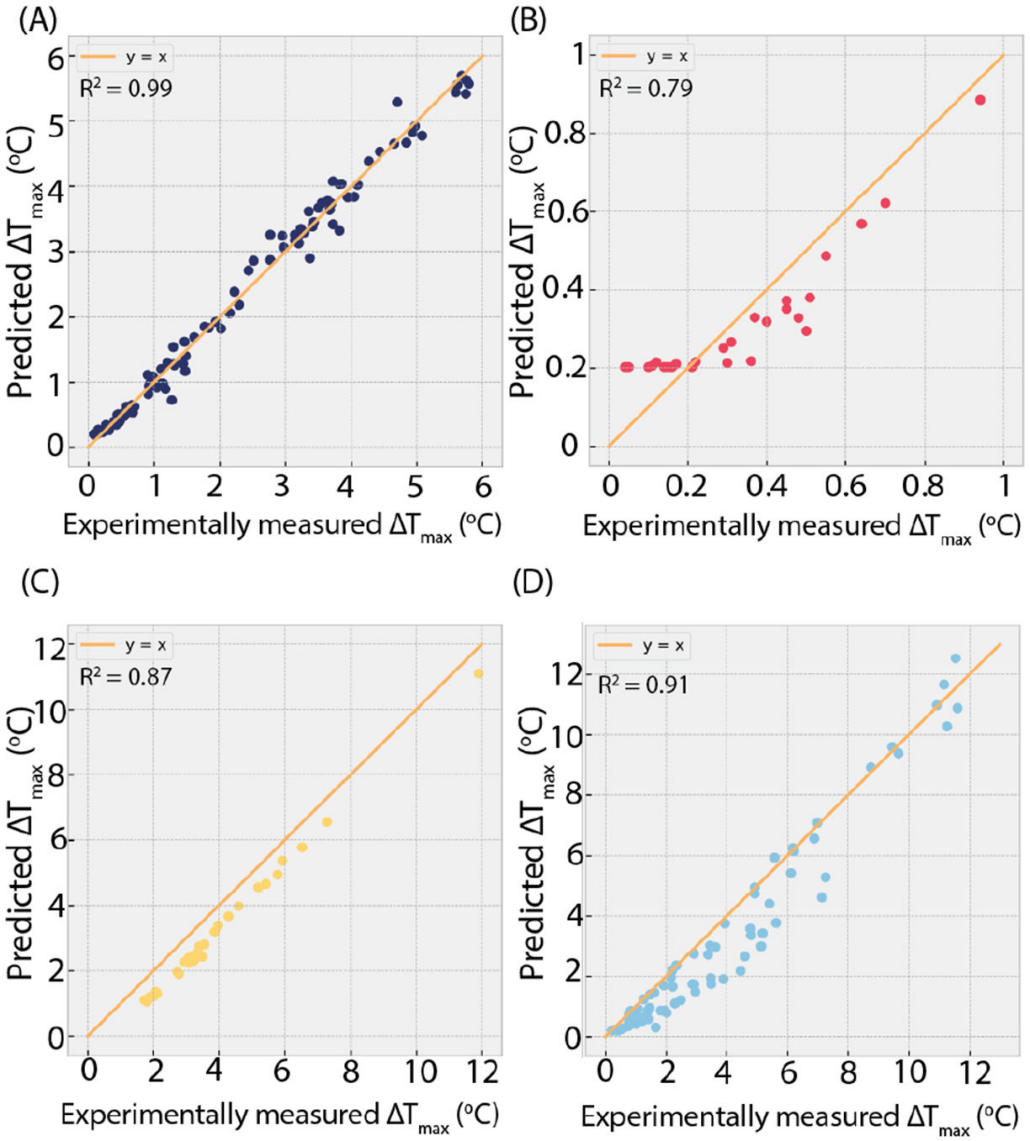
Performance of the neural network with (A) the initial hold-out test dataset, (B) DBS during MRI in an open-bore scanner at 1.2 T, (C) DBS during MRI at 1.5 T, and (D) CIEDs during MRI at 1.5 T.

The training and validation were completed after 100 epochs. The results showed that the NN was able to accurately predict the ΔT_max_ of DBS systems during 3 T MRI with a MSE of 0.32 °C^2^ and R^2^ of 0.99 for the hold-out test dataset. The results for the three additional test datasets were also promising, with MSEs of 0.01, 0.56, and 0.88 °C^2^ and R^2^ values of 0.79, 0.87, and 0.91 for RF heating of DBS systems during 1.2 T MRI and 1.5 T MRI and cardiac pacemaker systems during 1.5 T MRI, respectively. The comparison between the NN-predicted ΔT_max_ and the experimentally measured ΔT_max_ can be seen in Fig. 6. The training was completed within 1-3 minutes, and all predictions were performed in less than 1 minute.

## IV. Discussion & Conclusion

As the demand for MRI exams continues to grow, with an estimated 66-75% of patients with AIMDs such as DBS systems expected to require an MRI exam within 10 years of implantation [14], efforts to address the problem of RF heating have also increased. These efforts include modifying the material and design of leads [15], implementing new MRI transmit technology to establish a low electric field area tailored to the patient’s implanted lead [16]-[19], and investigating the use of ultra-high-field [20], [21] and open-bore vertical scanners [22]-[24]. Despite the ongoing efforts to mitigate RF heating in patients with AIMDs, MRI exams remain a challenge for these patients. Therefore, having real-time monitoring tools to predict RF heating of unknown implants on a personalized basis would be highly advantageous.

In this study, we proposed a ML-based solution to predict the temperature increase around a conductive lead. We designed a fully connected feedforward NN to estimate the ΔT_max_ using only the first five seconds of RF heating data. Our NN produced highly accurate predictions (R^2^ = 0.99) for the initial test dataset. Furthermore, we evaluated the performance of our NN under different field strengths, MRI scanner types, and AIMDs. The results indicated that the NN could successfully predict ΔT_max_ even with changes to the experimental conditions (R^2^ up to 0.91), except for some exceptional cases where the experimentally measured ΔT_max_ < 0.2 °C. This is crucial, as the configurations tested in our experiments reflected a wide range of lead trajectories and orientations with respect to the MRI electric fields, which can lead to substantial variability in the magnitude of RF heating, as observed in actual patients [25], [26].

Our future work aims to explore other ML architectures to compare the robustness of the present NN, improve the ability of the current NN to predict very low RF heating, and validate the ML-based predictions. Additionally, we will substitute the temperature probe data with data obtained from fast MR thermometry sequences, which have demonstrated their ability to accurately predict temperature increase in the presence of conductive leads [27].

## Clinical Relevance—

Machine learning-based algorithms can potentially enable rapid prediction of MRI-induced RF heating in the presence of unknown AIMDs in various MR environments.
